# Brain Single Photon Emission Computed Tomography Scan (SPECT) and functional MRI in Systemic Lupus Erythematosus Patients with Cognitive Dysfunction: A Systematic Review

**DOI:** 10.22038/aojnmb.2018.26381.1184

**Published:** 2018

**Authors:** Maryam Sahebari, Zahra Rezaieyazdi, Mandana Khodashahi, Bita Abbasi, Fazlollah Ayatollahi

**Affiliations:** 1 Rheumatic Diseases Research Center, Mashhad University of Medical Sciences, Mashhad, Iran; 2 Department of Radiology, Mashhad University of Medical Sciences, Mashhad, Iran

**Keywords:** Cognitive dysfunction, functional magnetic resonance imaging, SPECT, Systemic lupus erythematosus

## Abstract

**Objective(s):**

Systemic lupus erythematosus (SLE) is an autoimmune disease with a wide range of clinical manifestations. Cognitive dysfunction is one of the manifestations that could present prior to the emergence of any other neuropsychiatric involvements in SLE. Cognitive dysfunction is a subtle condition occurring with a high frequency. However, there is no data on the correlation of cognitive dysfunction with central nervous system (CNS) imaging findings, in particular single-photon emission computed tomography scan (SPECT) and functional MRI. We decided to perform a systematic review of brain SPECT and fMRI in SLE patients with cognitive dysfunction.

**Methods:**

PubMed, Scopus, and Google Scholar databases were searched until April 2017 with the following keywords: “SLE OR systemic lupus erythematous OR lupus” AND “functional MRI OR functional magnetic resonance imaging OR fMRI OR SPECT or SCAN”. A total of 1,767articles were found. Two rheumatologists reviewed the articles and finally 14 articles were selected for the final systematic review.

**Results:**

The fMRI and SPECT imaging techniques could provide valuable information regarding the SLE patients with cognitive dysfunction at the early stages of the disease.

**Conclusion:**

Brain SPECT scan and fMRI are used as functional imaging tools in SLE. Both of these diagnostic modalities are sensitive in reflecting the subtle brain damages in SLE patients with cognitive dysfunction. Brain fMRI and SPECT scan could be significantly beneficial in the diagnosis and initial management of cognitive dysfunction in SLE. Nevertheless, prospective studies could be useful in confirming the application of these diagnostic modalities in the clinical setting.

## Introduction

Among different neuropsychiatric manifestations of systemic lupus erythematosus (SLE), cognitive dysfunction has recently gained specific attention. Cognitive dysfunction is one of the inconspicuous manifestations of SLE that involves the central nervous system (CNS). A myriad of neuropsychiatric syndromes may occur at any time during the course of the disease. The patients inflicted with neuropsychiatric SLE (NP-SLE) usually complain of having problems in memory and executive skills such as attention, visual-spatial function, verbal fluency, motor function as well as planning, organizing, and sequencing information. 

The first study that investigated cognitive impairment in SLE patients reported a prevalence rate of 66% for this dysfunction among SLE patients. Cognitive dysfunction is the most common psychiatric manifestations of SLE, which is reported in up to 80% of SLE cases (1). It is often difficult to differentiate NP-SLE from other independent psychological issues. Moreover, the drug-induced cognitive dysfunction related to corticosteroids is another consideration in the diagnosis of NP-SLE. Therefore, in 1999, the American College of Rheumatology (ACR) provided nomenclature to determine the 19 neuropsychiatric syndromes seen in SLE (2).

Cognitive impairment in NP-SLE shows a strong relationship with hippocampal atrophy, which can be caused by autoantibodies (3), cytokines, hormones (4), and vascular pathologies such as microvasculopathy or thrombosis (5). Based on a study, the recently-diagnosed SLE patients had the greatest impairment in the Automated Neuropsychological Assessment Metrics (ANAM) subtests of cognitive efficiency, which requires sustained attention/vigilance, simple reaction time, and visuospatial span of attention/working memory (6). It seems that cognitive dysfunction could present in the initial course of the disease even before the emergence of any other neuropsychiatric manifestations. The evaluation of NP-SLE involves various laboratory and neuroimaging methods as well as neuropsychological assessments. Different neuroimaging modalities are used to detect structural and functional abnormalities such as tissue loss or atrophy, even at the level of the biochemical processes. These methods include computed tomography, magnetic resonance imaging (MRI), electroencephalogram, positron emission tomography, single-photon emission computed tomography (SPECT), and functional MRI (fMRI). 

Various brain pathologies may primarily represent as functional changes, preceding the anatomical changes. The brain SPECT provides three-dimensional (3D) functional information using a single gamma ray emission in addition to the injection of trace amounts of molecules labeled with a radioactive isotope. There are several studies indicating a correlation between disease activity and brain abnormalities on the SPECT in the parietal, frontal, and temporal lobes (7, 8).

Brain hypoperfusion in SLE may represent reversible lesions or subclinical CNS involvement. The SPECT imaging seems to be useful in detecting and monitoring CNS involvement in SLE. The fMRI is an MRI method that can detect brain functional impairments according to the changes in parenchymal perfusion. This technique is based on the relationship between neuronal function and blood flow. The fMRI can detect subtle alterations in brain perfusion occurring when the patient performs a task called paradigm. 

The standard technique for fMRI is based on the blood-oxygen-level dependence imaging that is based on the delicate autoregulatory mechanisms of the blood flow in the brain upon the activation of a brain area. In this regard, when an area in the brain is activated, the neuronal metabolism and oxygen consumption are increased. This initially leads to the elevation of deoxyhemoglobin concentration. After a gap of 2-6 sec, the autoregulation leads to the enhancement of the blood flow to the area, followed by the enhancement of oxyhemoglobin concentration. This oxyhemoglobin concentration makes the signal used in the fMRI for image acquisition.

Given the importance and high frequency of cognitive dysfunction, it seems essential to establish objective methods for the evaluation of cognitive problems in SLE. With this background in mind, the present systematic review was conducted to investigate the diagnostic value of fMRI and SPECT in SLE patients with cognitive dysfunction.

## Methods

***Literature search strategy***

An electronic literature review was conducted on the studies published until April 2017 in several databases including PubMed, Scopus, and Google Scholar. The searching process was performed using the following keywords: “SLE OR systemic lupus erythematous OR lupus” AND “functional MRI OR fMRI OR SPECT”. Any discrepancy between the electronic records was noted and resolved subsequently. In addition, all references of the selected articles were manually searched to find possible related articles. 

All peer-reviewed articles published in English and evaluated cognitive dysfunction in SLE patients by brain fMRI and/or brain SPECT was included. In addition, all different types of articles with various study designs including clinical trials, case-controls, cross-sectional research studies, cohort studies, and case series were eligible to be included.

The quality of the articles was assessed by the Oxford Center for Evidence-Based Medicine Checklist for diagnostic investigations (9). The selection of the studies and determination of quality score were performed by two independent investigators and the discrepancies were resolved by consensus. The disagreements between the two reviewers were resolved by obtaining one-third consensus. 

The SLE was defined using the 1982 ACR criteria (10) and cognitive dysfunction was defined based on the Diagnostic and Statistical Manual of Mental Disorders (DSM-IV) classification (11). 

The final decision was made to exclude the studies that contained the following criteria:

1. Inaccessible articles which could not be obtained by sending emails to the corresponding authors

2. A sample size of less than five patients

3. Conference papers and letters to the editor, review articles, and meta-analysis 

4. Animal studies 

***Study selection***

The initial study selection was made based on the titles and, where available, the abstracts in case of meeting the inclusion criteria. Subsequently, the full-text versions were obtained for the further assessment of the studies. The identification and removal of the duplicated references were performed in the next step. Some studies were added manually. 

**Figure 1.**
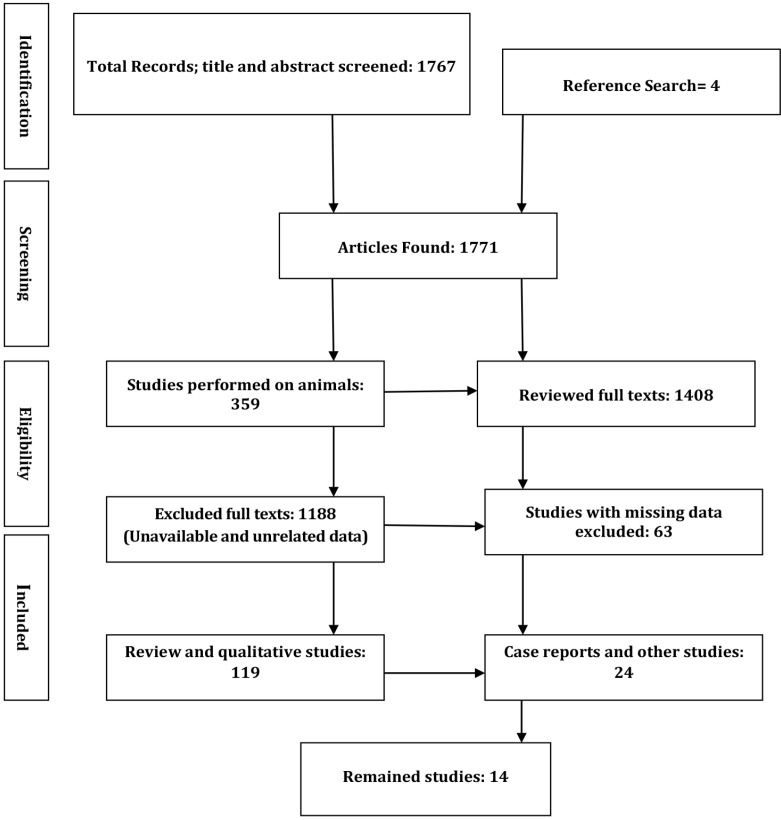
PRISMA flow diagram of the assessed studies

All human studies were included without any age limitation. A total of 1,767articles published until 2017 were collected based on their titles. Four additional articles were included by manually searching the reference lists of the previously selected papers. The title and abstract review resulted in the exclusion of 359 publications because animals were investigated. We included the articles that only evaluated the NP-SLE patients with cognitive dysfunction. Therefore, out of the remained titles, 14 potentially relevant publications were entered into the study after performing a step by step evaluation of the full-texts. The quality assessment of the included studies is presented in Table 1. Figure1 illustrates the PRISMA flow diagram of the studies assessed in this systematic review.

***Data analysis***

The evaluation of the articles was performed by two rheumatologists who appraised the selected articles independently. All available and necessary information, including the name of the first author, country and year of publication, sample size, intervention (i.e., SPECT or fMRI), and main findings were extracted. The third author was engaged in the data extraction process if needed. Due to the heterogeneity of the studies, a meta-analysis could not be performed in this review.

## Results

The summarized findings of the fourteen articles included in the final review are presented in Table 2. One study had been conducted on female patients with childhood-onset SLE (mean age: 17.3±3.4 years) (12). Ten articles implemented brain fMRI (12-21), while two articles performed only brain SPECT scan (22, 23). The other two articles used both brain SPECT and fMRI (24, 25). 

Shapira-Lichter et al. evaluated SLE patients without overt NP-SLE and reported that 3 out of 12 SLE patients suffered from cognitive dysfunction, including difficulty in remembering names and words (14). The reviewed studies utilized different diagnostic methods for the detection of cognitive dysfunction. This issue might limit the equal evaluation of all aspects of such a dysfunction. Moreover, 8 cases out of the 14 included articles employed control groups (13, 14, 16, 17, 19-22). Consequently, it would be impossible to compare the overall outcome in the presence of the studies having no control groups (15, 16, 18, 23-27).

## Discussion

Cognitive dysfunction is an important neuropsychiatric manifestation in SLE. It is difficult to diagnose cognitive dysfunction secondary to pure brain involvement in SLE patients due to insufficient knowledge of the exact pathophysiology and lack of approved standard objective tests. The current systematic review was focused on the studies utilizing brain fMRI and SPECT techniques for the assessment of cognitive dysfunction in SLE.

**Table 1.** Quality assessment of the 14 included articles until 2017 which evaluated the role of brain SPECT and/or fMRI in cognitive dysfunction among systemic lupus eryhematosus patients

**First author (reference number)**	**Randomized **	**Blinded**	**Withdrawals**	**Jadad score **
Driver et al. (22)	Uncertain	Yes	Uncertain	1
Zhang et al. (24)	Yes	Yes	Uncertain	2
DiFrancesco et al. (12)	Uncertain	Uncertain	Uncertain	0
Oh DH et al.(23)	Uncertain	Uncertain	Uncertain	0
Shapira-Lichter et al. (14)	Yes	Uncertain	Yes	2
Waterloo et al. (25)	Uncertain	Uncertain	Uncertain	0
Fitzgibbon et al. (13)	Uncertain	Uncertain	Uncertain	0
Nishimura et al. (21)	Uncertain	Uncertain	Yes	1
Ichinose et al. (20)	Uncertain	Uncertain	Uncertain	0
Sarbu et al (19)	Uncertain	Yes	Yes	2
Zimmermann et al. (18)	Uncertain	Uncertain	Uncertain	0
Cesar et al. (17)	Uncertain	Uncertain	Uncertain	0
Piga et al. (16)	Yes	Yes	Yes	3
Al-Obaidi et al. (15)	Uncertain	Yes	Uncertain	1

**Table 2:** Summary of the 14 articles which evaluated cognitive dysfunction in patients with neuropsychiatric systemic lupus erythematosus using brain functional MRI and/or SPECT

**First author** **(reference number), country/year**	**Sample size**	**Intervention**	**Main findings in patients with cognitive dysfunction**	**Examined paradigms**	**Imaging findings**
Driver et al. (22), USA/2008	37 NP-SLE^1^ patients, 19 normal controls (mean age: 42 years)	^99m^Tc-ECD^2^ brain SPECT^3^	30 (81.1%) cases out of 37 NP-SLE patients had abnormal scans	Cognitive dysfunction	The scans revealed markedly decreased perfusion in the watershed areas of the frontal lobes bilaterally. Severe cognitive dysfunction was associated with severe perfusion deficits, compared to mild cognitive dysfunction.
Zhang et al. (24), China/2005	43 patients with SLE (22 cases with NP-SLE and 21 cases with non-NP-SLE)	^99m^Tc-ECD brain SPECT, MRI^4^	6 cases out 7NP-SLE patients with cognitive dysfunction had abnormal SPECT	Cognitive dysfunction	19 (95.0%) out of 20 abnormal SPECT scans showed moderate to severe perfusion defect, mostly affecting multiple regions (17/20), especially in the frontal and parietal lobes and the basal ganglia (15, 11, 11, and 3 cases in the frontal, parietal, basal ganglia, and temporal lobes, respectively). In the NP-SLE patients with diffuse presentations, SPECT revealed 16/18 versus 6/18 abnormalities in MRI.
DiFrancesco et al. (12), USA/2007	10 childhood-onset SLE patients (7 cases with cognitive dysfunction)	Brain fMRI^5^	Six out of 10 childhood-onset SLE patients showed a statistically significant increased activation of brain in special areas involved in the CPT^6^, n-back, and verb generation tasks	Attention, working memory, and language processing	Language: childhood-onset lupus patientsshowed greater activation in the Broca’s area and non-activation of the Wernicke’s area. Attention: SLE patients exhibited more extensive activation than control subjects in the large tracts of the fusiform gyrus and visual associative cortex with abnormal attention Memory: the patient group had greater activation in the hippocampus and prefrontal regions.
Oh et al. (23), Korea/ 2011	19 SLE patients (6 cases with memory impairment)	Brain SPECT	In NP-SLE patients, 6 out of 9 regions of interest had a greater activation during working memory activity	Cognitive function and memory function	Significant hypoperfusion was observed in the right precuneus in the patients with memory impairment, which was possibly due to the association between impaired intrinsic functional connectivity in the default network and memory impairments in SLE
Shapira-Lichter et al. (14), England/2013	23 SLE patients (12 cases without clinically overt neuropsychiatric manifestations and 11 matched healthy controls)	fMRI	3cases out of 12 SLE patients without overt NP symptoms complained of cognitive dysfunction manifested as a difficulty in remembering names and words	Learning and recall function	The anterior medial prefrontal cortex of the DMN^7^ appeared to be the only region where brain activity dynamics were altered during the learning process and within free-recall period attempts. A significantly different brain activation dynamics was demonstrated in the patients with SLE as compared to that in the healthy controls.
Waterloo et al. (25), Norway/2001	57 SLE patients	Brain SPECT and MRI	No association was detected between cognitive dysfunction and rCBF^8^ in NP-SLE patients	Different areas of cognition, such as memory, attention, language, visuospatial processing, psychomotor speed, and executive function	Abnormal global CBF was observed in 31 patients. 17 patients had focal lesions of reduced blood flow. 50% of the patients had generalized bilateral CBF reduction, mostly in the temporal or frontal lobes. The areas of hypoperfusion were most frequently in the frontal, temporal, and parietal regions. 31/56 patients (55 %) showed CBF global reduction of> 15%. Significantly reduced rCBF was seen in the superior part of the frontal,frontal inferior, parietal superior, parietal inferior,and temporal lobes of 32, 17, 13, 26, and 43 patients, respectively.
Fitzgibbon et al. (13), New Zealand/2008	27 female patients (9 SLE patients, 9 healthy controls. and 9 rheumatic arthritis controls)	Brain BOLD-fMRI^9^	Greater frontoparietal activation in NP-SLE patients during the memory task	Working memory	The NP-SLE patients had greater degrees of cortical activation (i.e., increased cortical blood flow to activated areas) at six of the nine studied ROIs^10^ during performing the memory tasking, which was significant in three regions, namely the posterior inferior parietal lobules of both hemispheres separately and combined and supplementary motor area (mid-line frontal lobe).
Nishimura et al. (21) , Japan/2016	43 corticosteroid-naive patients with SLE	MRI and EEG	16.7% and 41.7% of the patients with NIC hadabnormal brain MRI and EEG, respectively	NCI	Patients with SLE had lower scores than the control subjects for RAVLT trials, reflecting immediate recall and psychomotor speed. Lower psychomotor speed was concluded to result from reduction in corpus callosum volume or other white matter abnormalities.
Ichinose et al. (20), Japan/2015	32 NP-SLE patients	MRI	17 (53.1%) cases out of 32 patients had abnormal MRI findings	NCI	Cytokines/chemokines were significantly higher in the NPSLE patients, compared to the MS/NMO patients.
Sarbu et al. (19), UK/2015	108 patients with neuropsychiatric lupus	MRI	Brain abnormalities were detectedin 59.3% of the patients	Cognitive dysfunction	Approximately, WMH, especially in frontal and parieto-occipital regions, was observed in half of the patients; however, only focal WMH with low lesion burden was observed in patients with normal MRI.
Zimmermann et al. (18), Brazil/2017	40 patients with SLE	MRI and fMRI	47% of the patients had abnormal MRI findings	Cognitive dysfunction	A decreased volume was reported in the left hippocampus, amygdala, and the right hippocampus in the SLE patients with cognitive dysfunction.
Cesar et al. (17), USA/2015	23 patients with neuropsychiatric systemic lupus	MRI	---	Cognitive dysfunction	Increased T2 lesion number and volume as well as greater lesion attenuation in the left superior and a small portion of the right posterior corona radiata were observed in the patients with NPSLE.
Piga et al. (16),Italy/2015	30 patients with longstanding SLE	MRI and fMRI	53.3% of the patients had MRI abnormalities, and 80% of them showed abnormalities in the follow-up MRI	Cognitive dysfunction	mMSS, cerebral volume loss, and new ischemic parenchymal lesions were observed in some cases.
Al-Obaidi et al. (15), UK/2016	27 Childrenwith NP-SLE	MRI	Cognitive dysfunction was observed in 14.8% of the patients	Cognitive dysfunction and anxiety disorder	The majority of the patients had no MRI abnormalities despite the signs and symptoms of active NPSLE. T2-weighted imaging showed cortical grey matter lesions, brain atrophy, basilar artery territory infarction, and focal white matter hyperintensities in some cases.

NP-SLE= Neuropsychiatric systematic lupus erythematous; ECD= ethyl cysteinate dimer; CPT= Continuous performance task; NMO= Neuromyelitis optica; DMN= Default mode network; rCBF= Regional cerebral blood flow; BOLD-fMRI= blood-oxygen-level-dependent functional MRI; ROIs= Regions of interest; NIC= Neurocognitive impairment; mMSS= Modified MRI scoring system; NCI= Neurocognitive impairment; RAVLT= Rey Auditory Verbal Learning Test

***Brain single-photon emission computed tomography scan (SPECT)***

In a study conducted by Driver et al. on 37 SLE patients with cognitive dysfunction, only seven patients were reported to have normal brain SPECT scans (i.e., 2, 2, and 3 patients with mild, moderate, and severe cognitive dysfunctions, respectively). The patients with severe cognitive dysfunction had more severe perfusion deficits in the watershed areas of the bilateral frontal lobes compared to those with mild ones (22). 

In a study performed by Oh et al., brain SPECT scan facilitated the detection of a significant hypoperfusion in the right precuneus in SLE patients with memory impairment. Accordingly, they concluded that the analysis of the statistical parametric mapping of the brain SPECT could be a beneficial tool to evaluate the regional cerebral blood flow (rCBF) in SLE patients with memory impairment (23).

Nishimura et al. revealed an association between the neurocognitive impairment (NCI) and general SLE activity. The dominant pattern of NCI involves reduced psychomotor speed and notable deficits in attention, information processing, learning, memory, and executive function (21). It was shown that slow psychomotor speed had a significant association with rCBF in the parietal lobe; therefore, cerebral infarct affects psychomotor speed. On the other hand, executive dysfunction was reported to have a significant relationship with rCBF in the frontal lobe and influenced by age. 

Waterloo stated that none of the cognitive dysfunction domains were associated with regional or global CBF observed on brain SPECT scan. In addition, cerebral infarcts detected by MRI were associated with cognitive dysfunction (25). Maeshima et al. found no relationship between higher cortical dysfunction and brain CT findings (28). Moreover, Waterloo revealed no association between cognitive dysfunction and decreased rCBF identified by brain SPECT scan (25). Therefore, the brain SPECT scan might not contain additional information to confirm the effect of CBF on cognitive dysfunction (25), which was strengthened by an earlier study by Kao et al. (29). 

^99m^Tc hexamethyl-propylene-aminoxime (HMPAO) SPECT studies can be performed before and after methylprednisolone pulse therapy in NP-SLE patients. This technique might play an important role in determination of the effects of pulse therapy on rCBF (30). Another research also reported the usefulness of ^99m^Tc ethyl cysteinate dimer (ECD) brain SPECT in determination of rCBF changes after methylprednisolone pulse therapy (31).

Patients with NP-SLE had more frequent multifocal hypoperfused SPECT areas in the frontal and parietal lobes and higher rate of concordant abnormal MRI plus SPECT. It was concluded that SPECT and MRI combination could be more beneficial in NP-SLE (32). In another study, hypoperfusion did not have any relationship with age, duration of SLE, levels of anti-DNA antibodies, as well as C3 and C4 fractions. Moreover, the patients with more active clinical disease had significant cerebral hypoperfusion and more cumulative tissue damage (7).

***Brain functional magnetic resonance imaging (fMRI)***

The evaluation of fMRI findings in patients with NP-SLE demonstrated a higher normal-appearing white matter fractional anisotropy histogram peak height and more significant activations of the putamen, dentate nuclei, and primary sensorimotor cortex contralaterally (27). Different cognitive paradigms with variable regions of interest have been evaluated in the studies focusing on the fMRI findings in patients with SLE.

In a study conducted by DiFrancesco et al., the MRI data revealed a greater activation in the hippocampus and prefrontal regions among SLE children with cognitive dysfunction (12). Furthermore, Zimmermann et al. demonstrated that SLE patients with cognitive problems had lower volume of the left hippocampus, amygdala, and right hippocampus, compared to their counterparts without such problem (18). The performance of cognition may be affected by the local anatomic heterogeneity of the white matter damage.

Cesar et al. reported that patients with NP-SLE experience showed a considerable reduction in the white matter tract integrity. However, the decreased auditory-verbal memory was not associated with changes in the white matter integrity among these patients. In the mentioned study, the patients demonstrated increased number and volume of T2 lesion and enhanced lesion attenuation in the left superior and a small portion of the right posterior corona radiata (17).

Language deficit is one of the earliest deficiencies developing in neurological conditions. The prototypic language areas in the brain are Broca’s and Wernike’s areas, both of which are impaired in non-symptomatic SLE patients (28). In this regard, DiFrancesco et al. observed more pronounced activation in the Broca’s area and less activation in the Wernike’s area during verb generation in SLE. This may represent the development of different neuralcircuits in SLE patients (12). Fusiform gyrus and visual associative cortex are believed to be associated with attention deficits (33, 34). Accordingly, DiFrancesco et al. observed more pronounced activation in this area (12).

Memory function is a complex entity, which involves many different brain areas. DiFrancesco et al. showed the greater activation of the hippocampus and prefrontal regions in SLE patients. This finding suggests the impairment of the areas associated with memory in these patients. They also suggested that impairment in the visual and attention areas mandates the memory task and makes the paradigm more impaired (12).

Fitzgibbon et al. also evaluated memory task and showed the greater activation of the frontoparietal regions. They hypothesized that these changes might be due to the impairment of the white matter circuits needed in memory task resulting in the generation of new pathways (13). Mackay et al. reported a significant difference in the brain activation patterns while performing memory and fearful face paradigms. They reported excessive cortical activity in SLE patients while performing a task and ascribed it to the recruitment of additional neuronal circuits, indicating the damaged state of the native circuits (26).

In another study performed by Piga et al., it was shown that SLE patients with and without NP-SLE had a significant brain damage progression which was associated with deterioration of the white matter hyperintensities, brain volume loss, and occurrence of new cortical parenchymal defects. Therefore, MRI-diagnosed brain damages are associated with a higher risk of developing neuropsychiatric manifestations (16). Inconsistent with the results of the other studies, in a research carried out by Al-Obaidi et al., brain MRI was normal in the majority of patients with SLE which was indicative of the fact that a normal brain MRI does not necessarily reject the presence of reversible NP-SLE (15). 

The cingulate gyrus performs an inhibitory function (35). DiFrancesco et al. showed that this area has more pronounced activation in control tasks among SLE patients (12). The brain SPECT scan was reported to be more sensitive than normal brain MRI (24, 29, 36, 37) in the detection of CNS involvement by many researchers. According to some studies, fMRI could be highly sensitive to assess the subtler cognitive dysfunction providing dynamic reflection of rCBF despite its limitation in showing the severity of cognitive deficits at rest (13). Nevertheless, regarding the various contradictory findings in this domain reported in different studies, further investigations are required to outline the valuable roles of these diagnostic modalities. 

## Conclusion

The brain diagnostic modalities including SPECT scan and fMRI are sensitive in the diagnosis of subtle brain damages at early stages of cognitive dysfunction in SLE. 
